# Blood Parasites and Health Status of Hibernating and Non-Hibernating Noctule Bats (*Nyctalus noctula*)

**DOI:** 10.3390/microorganisms10051028

**Published:** 2022-05-14

**Authors:** Petr Linhart, Hana Bandouchova, Jan Zukal, Jan Votýpka, Vojtech Baláž, Tomas Heger, Vendula Kalocsanyiova, Aneta Kubickova, Monika Nemcova, Jana Sedlackova, Veronika Seidlova, Lucie Veitova, Anton Vlaschenko, Renata Divinova, Jiri Pikula

**Affiliations:** 1 Department of Ecology and Diseases of Zoo Animals, Game, Fish and Bees, University of Veterinary Sciences, 61242 Brno, Czech Republic; linhartp@vfu.cz (P.L.); balazv@vfu.cz (V.B.); hegert4@gmail.com (T.H.); v.kalocsanyiova@seznam.cz (V.K.); kubickova-aneta@seznam.cz (A.K.); h18002@vfu.cz (M.N.); jsedlackova@vfu.cz (J.S.); seidlovav@vfu.cz (V.S.); h20328@vfu.cz (L.V.); pikulaj@vfu.cz (J.P.); 2 Department of Animal Protection and Welfare and Veterinary Public Health, University of Veterinary Sciences, 61242 Brno, Czech Republic; 3 Institute of Vertebrate Biology, Academy of Sciences of the Czech Republic, 60365 Brno, Czech Republic; zukal@ivb.cz; 4 Department of Botany and Zoology, Masaryk University, 61137 Brno, Czech Republic; rencazaj@gmail.com; 5 Department of Parasitology, Faculty of Science, Charles University, 12800 Prague, Czech Republic; jan.votypka@natur.cuni.cz; 6 Biology Centre, Institute of Parasitology, Czech Academy of Sciences, 37005 České Budějovice, Czech Republic; 7 Bat Rehabilitation Center of Feldman Ecopark, Lisne, 62340 Kharkiv, Ukraine; anton.vlaschenko@gmail.com

**Keywords:** *Babesia vesperuginis*, *Schizotrypanum*, *Chiroptera*, acid–base balance, haematology, blood chemistry, *Trypanosoma dionisii*, *Trypanosoma vespertilionis*

## Abstract

Co-existence of bats with a wide range of infectious agents relates to their co-evolutionary history and specific physiology. Here, we examined blood samples collected during hibernation and the post-hibernation period to assess the influence of trypanosomes and babesias on the health status of 50 Noctule bats (*Nyctalus noctula*) using nested PCR. The impact of blood parasites on health was assessed by analysis of haematology and blood chemistry parameters in 21 bats. Prevalence of trypanosomes (*Trypanosoma dionisii* and *T. vespertilionis*) and babesia (*Babesia vesperuginis*) was 44% and 8%, respectively. Analysis of blood parameters indicated impact of babesia on acid–base balance. Blood chemistry parameters showed a significant decrease in total dissolved carbon dioxide and bicarbonate, increased anion gap, and no change in blood pH, suggesting compensated metabolic acidosis. Adverse effects of babesia were only apparent in hibernating bats. Our results suggest differences in the pathogenicity of trypanosomes and babesia in bats. While trypanosomes in general had no significant impact on the health status, we observed alterations in the blood acid–base balance in *Babesia*-infected bats during hibernation. Despite being infected, *Babesia*-positive bats survived hibernation without showing any clinical signs.

## 1. Introduction

Bats represent a specific group of mammals in terms of the physiological adaptations associated with their lifestyle and the infectious agents they host. Bats have long been identified as reservoirs and hosts for a diverse range of viruses [1]. In addition to viruses, bats harbour many other pathogenic agents, including *Bartonella* spp. [2], *Borellia* spp. [3], *Serratia* spp. [4], *Pasteurella multocida* [5], enterobacteria [6], and *Histoplasma capsulatum* [7]. More than 30 *Trypanosoma* spp. are known to infect bats [8,9,10,11,12,13,14,15,16]. Among other blood parasites, babesias and haemosporidia of several genera (*Plasmodium*, *Hepatocystis*, *Nycteria*, *Polychromophilus*) were confirmed in bats [17,18,19,20,21], including the most recent findings in new geographic areas [22].

Co-existence of bats with a wide range of RNA viruses and many other infectious agents may originate from their specific physiology. Like birds, bats are capable of active flight and this ability is conditioned by high demands on energetic metabolism and oxygen consumption, resulting in a significantly high rate of oxidative stress as a selection pressure. High resistance to oxidative stress, when associated with infection, may help vertebrates capable of active flight in the arms races between hosts and their pathogens. As such, antioxidative mechanisms most likely contribute to the high degree of tolerance and/or resistance of bats to pathogens [23,24,25,26].

Hibernation is a specific physiological state that requires a variety of metabolic adaptations. In addition to markedly decreased heart and respiratory rates, body temperature, and renal function in hibernating mammals [27,28], this hypometabolic state is also associated with increased levels of ascorbic acid, which protects against oxidative stress [29], and nuclear factor erythroid-2-related factor (Nrf2) and superoxiddismutase activity, which protects against ischemia/reperfusion [30]. On the other hand, both natural and adaptive components of immune system functions decrease during hibernation, manifested by up to 90% decrease in total leukocytes in circulation, a lower complement level, decreased phagocytic capacity and cytokine production, and reduced lymphocyte proliferation and antibody production [31,32].

Hibernation, therefore, can play an important role in blood parasite infection as reduced immune functions may affect the bat’s ability to regulate the number of endoparasites. Hibernation also enables the spread of blood parasites, as some vectors continue to feed on bats during hibernation [33,34,35]. On the other hand, extreme conditions within the hibernator’s body may also affect the parasite’s metabolism, including its ability to multiply and survive within the host during the hibernation period. This can be parasite–host specific.

In infections such as rabies, virus multiplication was shown to slow down during hibernation [36]. A similarly negative impact of hibernation was observed in a study on marmots (*Marmota marmota*), where most intestinal parasites were eliminated or significantly reduced in hibernating animals [37]. In contrast, numbers of intestinal parasites in North American little brown bats (*Myotis lucifugus*) remained unaffected throughout their host’s hibernation, despite the body temperatures dropping close to 0 °C [38].

Little is known about the pathogenicity of trypanosomes and babesias, their prevalence in bat species throughout the year, their developmental cycles, vectors, and other characteristics of the infection cycle [12,39,40,41,42,43,44]. While bat-related *Babesia vesperuginis*–transmitted by *Argas vespertilionis* [43] or ixodid bat ticks [45]–is believed to be pathogenic to the host [17,43,46], bat trypanosomes from the subgenus *Schizotrypanum* (except for the *T. cruzi* complex) are transmitted by *Cimex* spp. [44] and considered non-pathogenic [47]. Adaptation of the parasite to host hibernation conditions is species-specific. As far as bat blood parasites are concerned, there are no studies on their presence in bats during hibernation or of their pathogenicity during this period. We hypothesised that hibernation modulates the impact of blood parasites on Noctule bats and that lowered pathogenicity allows for a higher prevalence of blood parasites. To address this hypothesis, we used hibernating *Nyctalus noctula* bats as a model and examined the prevalence and intensity of trypanosoma and/or babesia infections and associated pathogenicity reflected in haematology and blood chemistry parameters.

## 2. Materials and Methods

### 2.1. Animals

Blood samples were collected to assess the health status and to evaluate *Trypanosoma* spp. and *Babesia* spp. infections in 50 Noctule bats from a single colony submitted to a wildlife rescue centre after destruction of their natural hibernaculum in Pilsen, Czech Republic, in December 2015. After examination for injuries, 15 males and 35 females were overwintered in an artificial hibernaculum (temperature 7 °C; humidity >85%). Two months after admission to the rescue centre (February) and prior to release following hibernation (April/May), blood samples were taken and analysed for haematology and biochemistry parameters as a part of routine clinical examination, while polymerase chain reaction (PCR) was used to test for the presence of blood parasites. Prior to sampling, an overall condition assessment was performed on each bat.

### 2.2. Collection of Blood Samples and Haematology and Blood Chemistry Analysis

Blood samples for PCR analysis and blood smear examination were collected from all 50 bat individuals using methods described elsewhere [48,49]. Blood parameters were measured using the EC8+ cartridge on a VetScan i-STAT analyser (Abaxis, Union City, CA, USA). The measured parameters included pH value (pH), partial pressure carbon dioxide (pCO_2_, kPa), total carbon dioxide (tCO_2_, mmol/L), bicarbonate (HCO_3_, mmol/L), base excess (BE, mmol/L), sodium (Na, mmol/L), chloride (Cl, mmol/L), potassium (K, mmol/L), anion gap (AnGap, mmol/L), blood urea nitrogen (BUN, mmol/L), glucose (Glu, mmol/L), haematocrit (Hct, l/L), and haemoglobin (Hb, g/L). Blood smears were stained with Giemsa to assess relative leukocyte counts and number of blood parasites. For relative leucocyte counts, 100 white blood cells were determined in each blood smear. The blood smears were also used to assess intensity of infection, with the whole blood smear checked to assess primary infection status. In all *Trypanosoma*-positive smears, the total number of trypomastigotes was determined per blood smear due to the very low level of parasitaemia. In *Babesia*-positive smears, the percentage of infected erythrocytes was determined in 25 randomly chosen microscope fields per blood smear and the mean percentage of infected erythrocytes counted using the ImageJ utility [50]. Upon collection, and prior to post-hibernation release, bats were supplemented with fluids and energy by either oral or subcutaneous administration of glucose and saline solution. Blood samples were taken as a part of routine clinical examination. Each bat was handled in such a way as to minimise sampling distress, and blood sampling was performed in accordance with the Animal Ethics Procedures and Guidelines of the University of Veterinary Sciences Brno, Czech Republic.

### 2.3. Detection of Trypanosomes and Babesias in Blood Samples

Part of the blood sample collected as described above was left for subsequent DNA isolation and nested PCR diagnostics of trypanosomes and babesias. Total genomic DNA was isolated from the blood samples using a DNA isolation kit (High Pure PCR Template Preparation Kit, Roche, Basel, Switzerland), according to the protocol recommended by the manufacturer.

We used the nested PCR 18S rDNA analysis protocol for *Trypanosoma* spp. detection (~2100 bp) described previously by Seward et al. [51]. A nested PCR targeting the 561 bp fragment of babesia 18S rDNA was used to screen for the presence of *Babesia* spp., as described previously [52].

PCR was performed using the Mini Opticon (Bio-Rad, Hercules, CA, USA), with reactions undertaken in a 20 μL reaction mixture containing 10 μL 2× EmeraldAmp Max PCR Master Mix (Takara, Kusatsu, Japan), 4 μL water, 0.5 μL of each primer (10 pmol/μL) and a 5 μL aliquot of isolated DNA in the first round, and 5 μL of PCR product from the first round instead of DNA in the second round.

All DNA amplicons were sequenced, the sequences being edited and compared with GenBank database via Basic Local Alignment Search Tool (BLAST) search [53]. Representative sequences were deposited under GenBank acc. nos. MN046012, MN046111-MN046113, MN046116, MN046117 (18S rRNA).

### 2.4. Statistical Analysis

The Chi-square test was used to compare prevalence of blood parasites in males and females.

Normal distribution of variables was tested using the Kolmogorov-Smirnov and Shapiro-Wilk tests. All parameters were normally distributed, with the exception of BE in the hibernation dataset and all differential leukocyte count parameters. The cube root transformation was applied for the above-mentioned parameters, though normality was still not achieved. Finally, the paired data of the 21 bats sampled twice during captivity were used for the comparison of parameter development in individuals. We tested the influence of the three categorical predictor variables (sex, period, and infection status) and their interaction on variability in blood parameters using Simple Factorial ANOVA with Repeated Measures. Subsequent analysis of differences between blood parameter means influenced by infection status were compared separately for the hibernation and post-hibernation periods using the *t*-test for independent samples. Non-normally distributed parameters were tested using Kruskal-Wallis ANOVA. All analyses were performed in Statistica v.13.2.

## 3. Results

Sequencing of PCR products indicated that 22 of the 50 Noctule bats examined were positive for trypanosomes (18 cases of *Trypanosoma dionisii* and 4 cases of *T. vespertilionis*) and four were positive for *Babesia* *vesperuginis* (three co-infected by one of the above-mentioned trypanosomes). PCR-based prevalence of trypanosomes and babesia was 44% and 8%, respectively. A detailed overview of the total number of bats, males and females, and prevalence of individual blood parasites is summarised in Table 1. Blood smear-based prevalence of trypanosomes and babesia in hibernating bats was 26% and 6%, respectively (Table 2).

**Figure 1:**
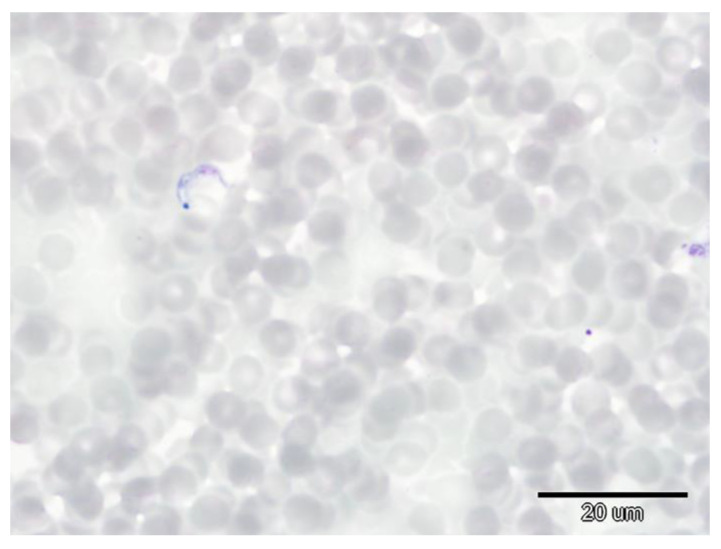
*Trypanosoma dionisii* in a blood smear from a Noctule bat female in hibernation (Giemsa staining, magnification of 1000×).

**Figure 2:**
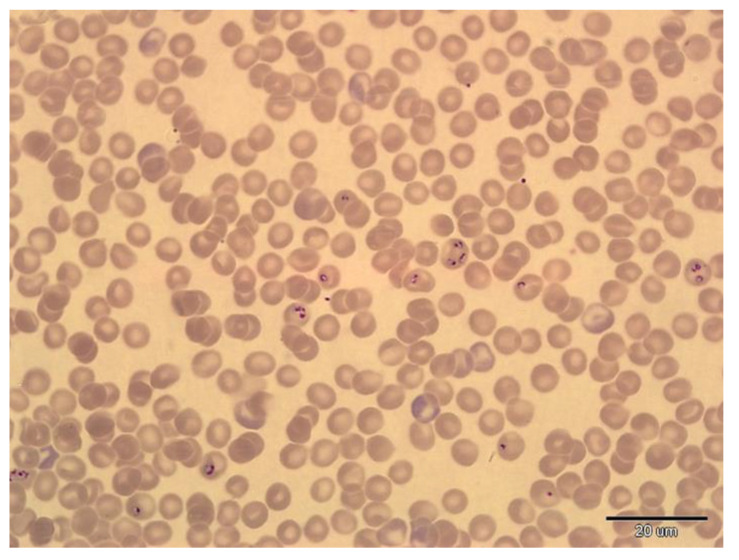
*Babesia vesperuginis* in a blood smear from a heavily infected Noctule bat female with acid–base imbalance in hibernation (Giemsa staining, magnification of 1000×).

Parasitaemia of *T. dionisii* and *T. vespertilionis* was very low during both periods. Only 13 bats proved positive during hibernation based on blood smears, with the total number of trypanosomes varying from 1 to 4 trypomastigotes per blood smear (Figure 1). Blood samples were re-checked by PCR during the post-hibernation period, the results indicating just one previously *Trypanosoma*-positive and one *Babesia*-positive bat as PCR negative. All blood smears from *Trypanosoma*-positive animals were microscopically negative during the post-hibernation period. Blood smear examination indicated a very high intensity of *B. vesperuginis* infection in one positive female (Figure 2), with the percentage of *Babesia*-infected erythrocytes per microscope field being 1.5 times higher during hibernation (4.4% compared with 2.85% post-hibernation). Very low parasitaemia (0.1%) was confirmed in two PCR *Babesia*-positive bats based on hibernation and post-hibernation blood smear examination, with one bat confirmed as *Babesia*-negative during hibernation based on blood smear microscopy. Results of haematology and blood chemistry parameters analysis can be found in an additional dataset file [see Supplementary Table S1: Blood_parameters_results].

We obtained complete paired results of blood parameters during both deep hibernation (February) and the post-hibernation period (April/May) from 21 (10 males and 11 females) of the 50 bats sampled. The bats were divided according to sex and infection status (10 males and 11 females, 13 negative, 5 *Trypanosoma*-positive and 3 *Babesia*-positive). These data were used to evaluate the effect of blood parasites on the health of bats during hibernation and post-hibernation periods.

**Figure 3:**
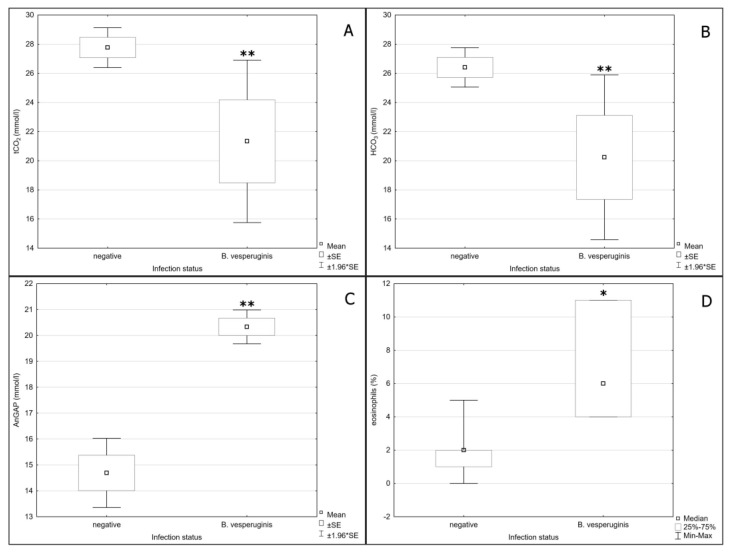
Differences of (**A**) total carbon dioxide values (negative–*Babesia*-negative, *B. vesperuginis*–*Babesia*-positive, *p* = 0.001589), (**B**) bicarbonate values (negative–*Babesia*-negative, *B. vesperuginis*–*Babesia*-positive, *p* = 0.002188), (**C**) anion gap values (negative–*Babesia*-negative, *B. vesperuginis*–*Babesia*-positive, *p* = 0.003313) and (**D**) eosinophils (negative–*Babesia*-negative, B. *vesperuginis*–*Babesia*-positive, *p* = 0.020801) in blood of a *Babesia*-positive bats in hibernation; * = *p* < 0.05, ** = *p* < 0.01.

The Chi-square test indicated distribution of parasite infection as randomly based, while Repeated Measures ANOVA confirmed that 7 of 12 haematological parameters changed in individual specimens between the first and second measurements. These differences were partly caused by physiological changes during hibernation; however, tCO_2_ and HCO_3_ were mainly influenced by infection status (Table 3), with *Babesia*-positive bats showing significant decreases during hibernation (Figure 3A, B). There was a highly significant increase in AnGap in *Babesia*-positive animals during hibernation, though the impact of infection was only confirmed by univariate analysis (Figure 3C). In the non-normally distributed parameters, Kruskal-Wallis ANOVA only indicated a significant difference with regards to an increase in relative eosinophil count in *Babesia*-positive bats during hibernation (Figure 3D), with no differences in either *Trypanosoma*-positive or *Babesia*-positive bats during the post-hibernation period.

## 4. Discussion

Trypanosomes and babesias are important infectious agents causing human and animal diseases that can be associated with mortality [54,55]. In this study, we examined blood samples of 50 Noctule bats originating from one hibernation colony. Analysis for blood parasites identified *B. vesperuginis* and the trypanosomes *T. dionisii* and *T. vespertilionis*, both of the subgenus *Schizotrypanum*, all of which have previously been described in European bat species. However, no previous study examined such a large group of hibernating individuals of a single bat species from one colony, monitored the bats for several months during hibernation, or examined haematological and blood chemistry parameters.

Based on the study of Gardner et al. [42], overall prevalence of the subgenus *Schizotrypanum* in 12 bat species in the UK was 17%, with a prevalence of 33% in Suffolk (southern England) and 0% around Inverness (Scotland). Gardner et al. [42] used thick and thin blood film microscopy to determine presence of trypanosomes; hence, we can expect a degree of underestimation compared with nested PCR diagnostics. We recorded an overall PCR prevalence of the subgenus *Schizotrypanum* of 44%, with prevalence of individual trypanosomes significantly in favour of *T. dionisii* (36%), compared with just 8% *T. vespertilionis* (Table 1). In comparison, Hamilton et al. [12] recorded 3 of 8 Noctule bats from Bristol, UK, as *T. dionisii*-positive, with one co-infected with *T. vespertilionis*.

Prevalence of *B. vesperuginis* in our study was 8%, compared with a total prevalence of 4% in the study of Gardner et al. [42]; though again, these results are based on microscopic examination of blood smears. Corduneanu et al. [19] recorded a PCR prevalence of *B. vesperuginis* of 4.3% in 24 bat species from the Czech Republic, Romania, and Austria, but 9.1% in Noctule bats from Brno (Czech Republic).

While no significant changes in blood parameters were observed in *Trypanosoma*-positive individuals during the hibernation and post-hibernation periods, we found significant changes in acid–base balance and higher relative numbers of eosinophils in blood samples collected from *Babesia*-positive individuals during hibernation. We observed a decrease in HCO_3_ and pCO_2_ and an increase in AnGap in these hibernating animals, with no change in pH, suggesting compensated metabolic acidosis, probably due to an increase in lactate levels caused by tissue hypoxia [56]. These findings correspond to changes in the acid–base balance found in dogs with fatal babesiosis due to *Babesia canis rossi* infection [57]. Likewise, severe metabolic acidosis was found in two reindeer infected with *B. odocoilei*, one of which died because of the infection [58]. While both the dogs and reindeer suffered acute uncompensated acidosis, we recorded compensated acidosis in the bats examined here. Thus, we can assume that these changes reflect the chronic phase of infection. This is supported by the absence of any decrease in haemoglobin levels in our *Babesia*-positive bats. Such a decrease was described by Gardner and Molyneux [43] during the acute phase of infection in common pipistrelle bats (*Pipistrellus pipistrellus*) experimentally infected with *B. vesperuginis*. In their study, parasitaemia reached a peak within 40 days post-infection and subsequently decreased significantly over the next 14–21 days. This decrease in haemoglobin correlated with the development of parasitaemia, and then returned to normal when the number of babesia in the blood decreased [43]. Importantly, the bats were active in the experiments of Gardner and Molyneux [43], hence their immune system was unaffected by hibernation. The differences in relative eosinophil number recorded in our *Babesia*-positive bats do not correspond with results for *Babesia*-positive Madagascan fruit bats (*Pteropus rufus*), for which no differences were observed between positive and negative animals [20], probably due to very low parasitaemia. When we examined the number of babesia in blood smears of four hibernating *Babesia*-positive animals, we found very high parasitaemia in one (4.4% of infected erythrocytes/field). Surprisingly, no differences in blood parameters were recorded between *Babesia*-positive and -negative animals in the post-hibernation period. Additionally, numbers of babesia in a blood smear from a highly positive bat were reduced during the post-hibernation period (2.85% of infected erythrocytes/microscope field). Hibernation, therefore, appears to play an important role in the impact of *B. vesperuginis* infection in bats, probably because of the bats reduced immune response and an inability to control babesia numbers in the blood. The overall influence of babesia on the bat is probably regulated, however, by restored immune function post-hibernation.

Unfortunately, we do not know what stage of babesia infection our bats were in before entering hibernation; however, it appears that hibernation allows prolonged parasitaemia, increasing thus the chance of spread to other hosts through vectors. Possible vectors of trypanosomes and babesias have been identified in hibernating bats [33]. *Ixodes vespertilionis* is known to feed mainly on bats during hibernation [34]. Hibernating bats appear to be able to handle long-term infections, despite their immune system being unable to fully respond to the babesia in their blood. Thus, our results only partly confirm the assumptions of previous studies [42,43], i.e., that *B. vesperuginis* is pathogenic to bats. Rather, we incline to the opinion of Ranaivoson et al. [20], who stated that the widespread host and geographic range of *B. vesperuginis* suggests that pathology associated with *B. vesperuginis* infection is unlikely to be severe in bats. The only case of *B. vesperuginis* related mortality previously described in a bat is that related to a pregnant female pipistrelle bat heavily co-infected with *Schizotrypanum* spp. [43]. In the same study, even intraperitoneally *Babesia*-infected pipistrelles were able to overcome the infection. Clearly, the influence of babesia on bat health in our study was more pronounced than the effect of trypanosome infection, but only during hibernation. In the post-hibernation period, however, the impact of babesia diminished and differences in blood parameters disappeared.

A possible explanation for low pathogenicity of trypanosomes and babesias in bats may be related to the host-pathogen-vector relationship and the life strategy of bat ectoparasites. In bats, transmission of these blood parasites is vector-dependent. In the case of *T. dionisii* and *T. vespertilionis*, the probable vectors are the bat bug *Cimex pipistrelli* or the bed bug *C. lectularius* [8], while it is the soft tick *Argas vespertilionis* [43] or the hard ticks *Ixodes ariadnae* and *I. vespertilionis* [45] in the case of *B. vesperuginis*. All of these blood-sucking arthropods are nidicolous [59,60], minimally mobile over longer distances (moving from one place to another on their host’s body [61]), and able to survive long periods without a host. As such, they are extremely dependent on their host repeatedly visiting the same shelters as bats alternate roosting sites during the year. As Noctule bats mainly use tree cavities or house facades as shelters, a fatal acute infection would mean a dead end for these blood parasites as bat shelter fidelity would greatly reduce the chances of moving to another host.

To avoid a strong immune system reaction, *T. cruzi* complex trypanosomes minimise their negative effects on the host by postponing the acute phase of infection escaping to the tissues. This strategy allows them to survive in the host throughout its life and results in life-long intermittent low parasitaemia in the infected host [62]. Similarly, babesias are also able to escape from the host’s immune response, thereby causing low parasitaemia and ensuring efficient transmission to another vector [63]. Hence, babesias and trypanosomes probably both share the same success strategy in bats and any accidental negative impact on the host probably represents an unwanted effect. On the other hand, trypanosomes of the subgenus *Schizotrypanum* are known to form cystic structures in bat organs and tissues, including the heart and skeletal muscles [64], and this was confirmed in *T. cruzi* also [62,65]. Thus, chronic effects involving tissue damage caused by the host’s own antibodies targeting encysted developmental trypanosome stages, similar to the Chagas disease [66], may be expected in bats during long-term infection [67]. Would this affect the population of a particular species? The influence of blood parasites cannot be ignored. Future studies need to examine co-exposure to other stressors and long-term effects in a greater detail, especially in the case of bat trypanosomes.

## 5. Conclusions

In this study, we confirmed differences in pathogenicity of trypanosomes and babesia in bats. Trypanosomes had no significant impact on blood parameters or the overall health status, but were more prevalent in the Noctule population examined. In *Babesia*-positive bats we found metabolic acidosis at similar levels previous studies described in mostly fatal cases of babesiosis in other vertebrate species. These differences were only apparent in samples collected during hibernation. There was no difference in acid–base parameters between *Babesia*-positive and -negative bats during the post-hibernation period, and infected bats survived several months with no sign of infection. Thus, hibernation may play a role in worsening the course of babesiosis in bats, probably due to a weakened immune response controlling the number of babesia in blood. Both trypanosomes and babesias are able to survive within the host, even under hibernation conditions. In general, we can say that while babesia caused chronic metabolic acidosis in hibernating bats in our study, neither trypanosomes nor babesia caused serious fatal infections. On the other hand, individual responses and potential combined exposures to other stressors should also be taken into account, especially during hibernation. Further investigations should be focused on in vitro studies of interactions between blood parasites and primary bat cell lines, chronic changes in target tissues, and antibody responses, especially in the case of trypanosomes.

## Figures and Tables

**Table 1:** Total PCR-based prevalence of blood parasites and prevalence in males and females.

Sex	No.	*B. vesperuginis*	*T. dionisii*	*T. vespertilionis*
Female	35	8.6%	40.0%	8.6%
Male	15	6.7%	26.7%	6.7%
Total	50	8.0%	36.0%	8.0%

**Table 2:** Total blood smear-based prevalence of blood parasites and prevalence in males and females.

Sex	No.	*B. vesperuginis*	*T. dionisii*	*T. vespertilionis*
Female	35	5.7%	28.6%	0%
Male	15	6.7%	20.0%	0%
Total	50	6.0%	26.0%	0%

**Table 3:** Repeated Measures ANOVA in parameters influenced by infection status.

Parameter	Infection Status	Measures	Sex	Change
tCO_2_	F = 6.209/*p* = 0.011	F = 4.945/*p* = 0.042	-	↓
HCO_3_	F = 6.052/*p* = 0.018	F = 5.863/*p* = 0.028	-	↓

## Data Availability

Not applicable.

## Supplementary Materials

The following supporting information can be downloaded at: https://www.mdpi.com/article/10.3390/microorganisms10051028/s1, Table S1: Blood_parameters_results.
